# Correction: Ziemssen et al. Immune Response to Initial and Booster SARS-CoV-2 mRNA Vaccination in Patients Treated with Siponimod—Final Analysis of a Nonrandomized Controlled Clinical Trial (AMA-VACC). *Vaccines* 2023, *11*, 1374

**DOI:** 10.3390/vaccines12080911

**Published:** 2024-08-12

**Authors:** Tjalf Ziemssen, Marie Groth, Veronika Eva Winkelmann, Tobias Bopp

**Affiliations:** 1 Department of Neurology, Center of Clinical Neuroscience, Carl Gustav Carus University Clinic, University Hospital of Dresden, Technische Universität Dresden, 01307 Dresden, Germany; 2 Novartis Pharma GmbH, 90429 Nuremberg, Germany; marie.groth@novartis.com (M.G.); veronika.winkelmann@novartis.com (V.E.W.); 3 Institute for Immunology, University Medical Center of the Johannes Gutenberg University, 55131 Mainz, Germany; boppt@uni-mainz.de

The authors would like to make the following corrections to this published paper [1].

There was a mistake in the legend and picture for Figure 1. For month 6 for the cohort “siponimod continuous”, one patient’s data were missing. This also affected the number of booster patients with identical month 6 and month 1 after the booster visit in the figure legend (old: 10 booster patients with n = 6 in cohort 1; corrected: 11 booster patients with n = 7 in cohort 1). The correct legend and figure appear below.

There was a mistake in the legend and picture for Figure 2. The number of booster patients with identical month 6 and month 1 after the booster visit was wrong (old: 10 booster patients with n = 6 in cohort 1; corrected: 11 booster patients with n = 7 in cohort 1). The labels of the *x*-axis of Figure 2A for month 1 after the booster in the cohort “siponimod continuous” contained an error (old: n = 16; corrected: n = 14). Furthermore, in the analysis of total anti-spike antibodies (Figure 2B), patients with an invalid control had been erroneously included. This has been corrected in accordance with an amendment to the clinical study report. The correct legend and figure appear below.

There was a mistake in the legend and picture for Supplementary Figure S1. In Figure S1A, one patient’s data were missing at month 6 in the cohort “siponimod continuous” and have since been added. This change also affected the figure legend: the explanation on the missing data (*) had to be revised (old: *one sample missing for month 1 and month 6; corrected: *one sample missing for month 1), as well as the number of booster patients with identical month 6 and month 1 after the booster visit (old: 10 booster patients with n = 6 in cohort 1; corrected: 11 booster patients with n = 7 in cohort 1). Furthermore, the explanations on missing data in Figure S1B (*, **) were not clearly assigned to part (B) of the figure legend. The explanations have been moved up. The correct legend and figure appear below.

**Figure 1:**
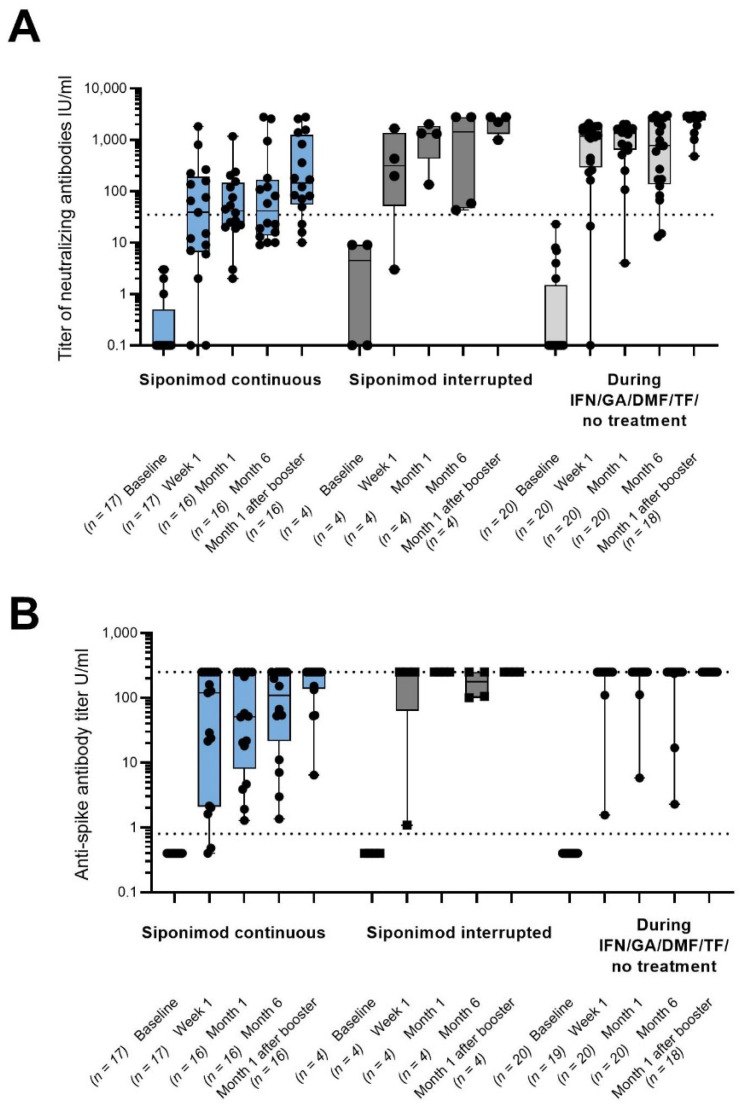
(**A**) SARS-CoV-2-specific neutralizing antibody levels in U/mL. (**B**) SARS-CoV-2-specific serum total antibody levels in U/mL. All the patients with available data were included in the analysis, and individual values are represented by dots. For 11 booster patients, the month 6 visit and the month 1 after the booster visit were identical (cohort 1: n = 7; cohort 2: n = 1; cohort 3: n = 3). The bars show the median values; the black dotted lines indicate assay-specific cut-offs for seropositivity; and the gray dotted lines indicate the maximal value of the quantification range. DMF: dimethyl fumarate; GA: glatiramer acetate; IFN: interferon-beta; n: number of patients with assessments; TF: teriflunomide; and U: units.

**Figure 2:**
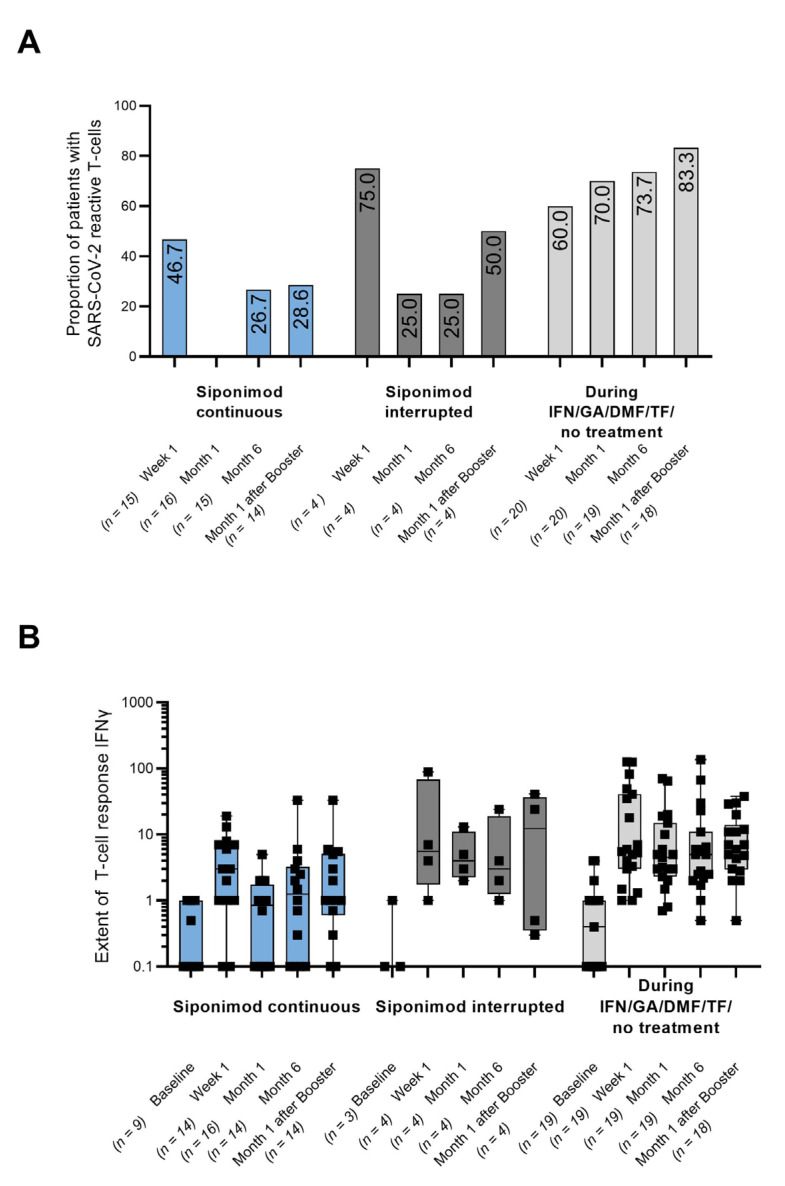
(**A**) T-cell response defined as the presence of SARS-CoV-2-reactive T-cells measured by the secretion of either IFN-, IL-2, or both (any level above basal activity); (**B**) ELISpot-based quantification of T-cell reactivity by calculation of IFN- stimulation indices towards SARS-CoV-2. Each dot represents one patient, and the medians are indicated by horizontal lines. DMF: dimethyl fumarate; GA: glatiramer acetate; IFN: interferon-beta; IFN-: interferon-; n: number of patients with assessments; PBMC: peripheral blood mononuclear cell; and TF: teriflunomide. The T-cell response could not be assessed in three patients with the continued siponimod treatment, one patient in the control group at the month 6 visit, and two patients of cohort 3 at month 1 after the booster because of insufficient cell counts after PBMC isolation. For 11 booster patients, the month 6 visit and the month 1 after the booster visit were identical (cohort 1: n = 7; cohort 2: n = 1; cohort 3: n = 3).

**Figure S1:**
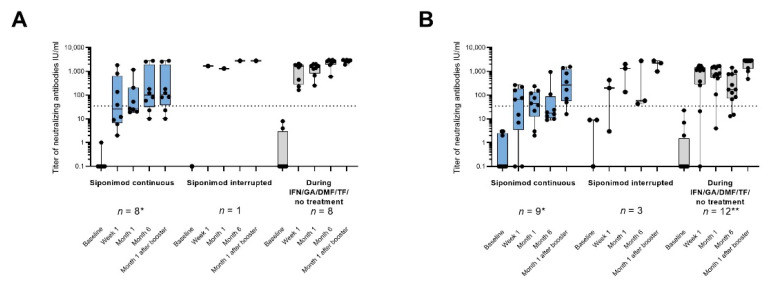
SARS-CoV-2-specific neutralizing antibody levels in U/mL by the timing of booster vaccination. (**A**) Booster vaccination before month 6; * one sample missing for month 1. (**B**) Booster vaccination after month 6; * one sample missing for month 6 and month 1 after booster; and ** two patients did not receive a booster vaccination. All the patients with available data were included in the analysis, and the individual values are represented by dots. For 11 booster patients, the month 6 visit and the month 1 after booster visit were identical (cohort 1: n = 7; cohort 2: n = 1; cohort 3: n = 3). The bars show the median values, and the black dotted lines indicate the assay-specific cut-off for seropositivity. DMF: dimethyl fumarate; GA: glatiramer acetate, IFN: interferon-beta; IU: international units; n: number of patients with assessments; and TF: teriflunomide.

The proportion of patients with SARS-CoV-2-specific T-cell responses in cohort 1 mentioned within the text (Abstract and Results) was inconsistent with the data in Figure 2A (old text version: 28.5%; corrected text version: 26.7%). The original sentence in the Abstract, “T-cell responses were seen in 28.5%, 25.0%, and 73.7% at month 6 and in 28.6%, 50.0%, and 83.3% after the booster (cohorts 1, 2, and 3, respectively)”, should be changed to “T-cell responses were seen in 26.7%, 25.0%, and 73.7% at month 6 and in 28.6%, 50.0%, and 83.3% after the booster (cohorts 1, 2, and 3, respectively).” The first sentence in Paragraph 3, Results Section, “SARS-CoV-2-specific T-cell responses were seen in only 28.5% (cohort 1) and 25.0% (cohort 2) at month 6, as well as in 28.6% (cohort 1) and 50.0% (cohort 2) at month 1 after the booster”, should be updated to “SARS-CoV-2-specific T-cell responses were seen in only 26.7% (cohort 1) and 25.0% (cohort 2) at month 6, as well as in 28.6% (cohort 1) and 50.0% (cohort 2) at month 1 after the booster”.

The authors state that the scientific conclusions are unaffected.

These corrections were approved by the Academic Editor. The original publication has also been updated.
