# Supplementary material for: Immune Response to Initial and Booster SARS-CoV-2 mRNA Vaccination in Patients Treated with Siponimod—Final Analysis of a Nonrandomized Controlled Clinical Trial (AMA-VACC)
Source: Vaccines (Basel). 2023 Aug 16;11(8):1374. doi: 10.3390/vaccines11081374 (PMC10459882; doi:10.3390/vaccines11081374)
Supplement: Supplementary file 1 [file vaccines-11-01374-s001.zip › vaccines-2522764-supplementary.pdf]

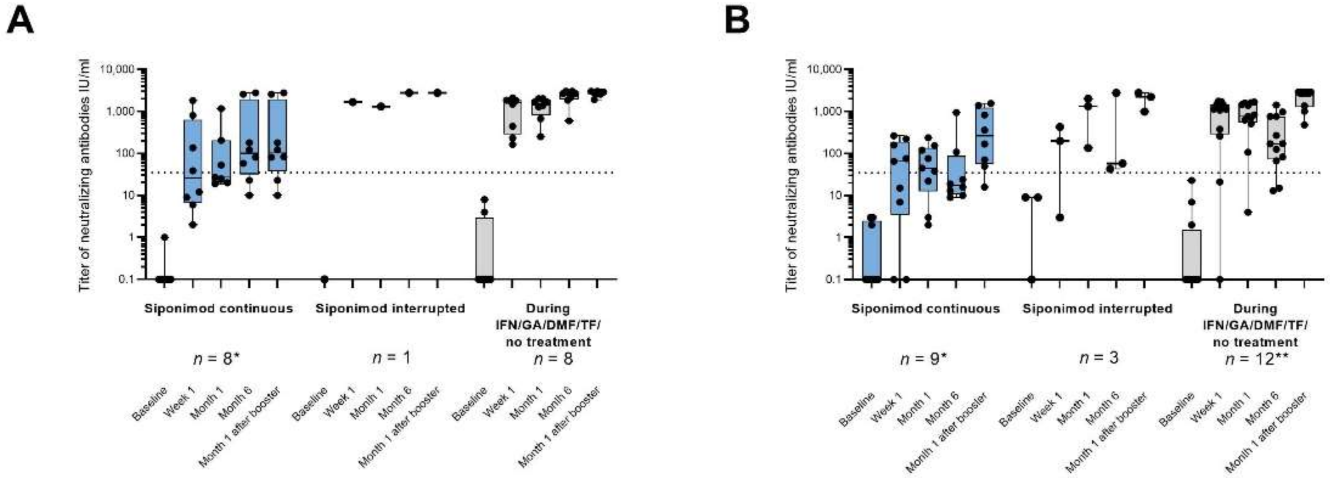

Figure S1. SARS-CoV-2-specific neutralizing antibody levels in U/mL by the timing of booster vaccination. (**A**) Booster vaccination before month 6; * one sample missing for month 1. (**B**) Booster vaccination after month 6; * one sample missing for month 6 and month 1 after booster; and ** two patients did not receive a booster vaccination. All the patients with available data were included in the analysis, and the individual values are represented by dots. For 11 booster patients, the month 6 visit and the month 1 after booster visit were identical (cohort 1: n = 7; cohort 2: n = 1; cohort 3: n = 3). The bars show the median values, and the black dotted lines indicate the assay-specific cut-off for seropositivity. DMF: dimethyl fumarate; GA: glatiramer acetate, IFN: interferon-beta; IU: international units; n: number of patients with assessments; and TF: teriflunomide.
